# Pediatric menisci: normal aspects, anatomical variants, lesions, tears, and postsurgical findings

**DOI:** 10.1186/s13244-024-01867-6

**Published:** 2024-12-12

**Authors:** Flávia Ferreira Araújo, Júlio Brandão Guimarães, Isabela Azevedo Nicodemos da Cruz, Leticia dos Reis Morimoto, Alípio Gomes Ormond Filho, Marcelo Astolfi Caetano Nico

**Affiliations:** Department of Musculoskeletal Radiology, Fleury Medicina e Saúde Higienópolis, São Paulo, Brazil

**Keywords:** Meniscus, Discoid meniscus, Meniscal injuries

## Abstract

**Abstract:**

The reported incidence of meniscal tears in the pediatric age group has increased because of increased sports participation and more widespread use of MRI. Meniscal injury is one of the most commonly reported internal derangements in skeletally immature knees and can be associated with early degenerative joint disease leading to disability. The pediatric meniscus has particularities, and knowledge of normal anatomy, anatomical variations, and the patterns of meniscal injury in the pediatric age group is essential to provide a correct diagnosis.

**Critical relevance statement:**

Accurate MRI interpretation of pediatric meniscal injuries is crucial. Understanding age-specific anatomy, vascularity, and variations can improve diagnostic precision, guiding targeted treatments to prevent early joint degeneration and disability.

**Key Points:**

- Meniscal lesions are common injuries in skeletally immature knees.
- Awareness of anatomical meniscus variants, patterns of injury, and associated injuries is essential.
- Meniscal tears in pediatric patients should be repaired if possible.

**Graphical Abstract:**

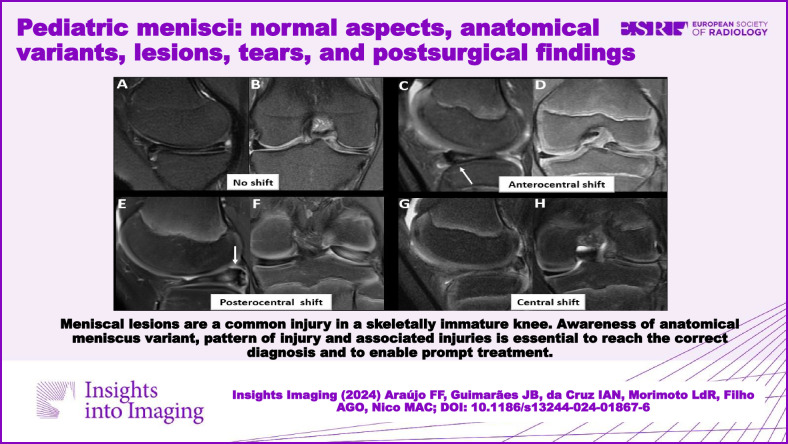

## Introduction

Meniscal injuries are some of the most common knee injuries in pediatric patients, together with anterior cruciate ligament (ACL) lesions [1, 2]. Although meniscus tears are less prevalent in the pediatric population than in adults, the reported incidence is increasing over time because of more intense sports activity in younger patients, with an overall meniscal injury rate of 5.1 per 100,000 athletes [3, 4].

Recognition and treatment of meniscal injuries are highly important because the meniscus is essential for joint mechanics and cartilage protection. This importance is particularly emphasized in younger athletes, as meniscal injuries and surgical removal can result in significant long-term consequences [5].

Careful consideration is warranted during the assessment of pediatric meniscus imaging, as the application of adult imaging principles to children may yield diagnostic inaccuracies. Such errors may contribute to adverse prognostic outcomes, prolonged recovery periods for resuming sports activities, and potential legal ramifications. Radiologists must possess a comprehensive understanding of the anatomical features and nuances of the pediatric meniscus to mitigate the likelihood of such errors.

Therefore, the purpose of this manuscript is to review the anatomy, variations, developmental changes, meniscal injuries, and postoperative imaging aspects. These findings are vital for enabling radiologists to make accurate diagnoses.

## Meniscal anatomy

**Fig. 1:**
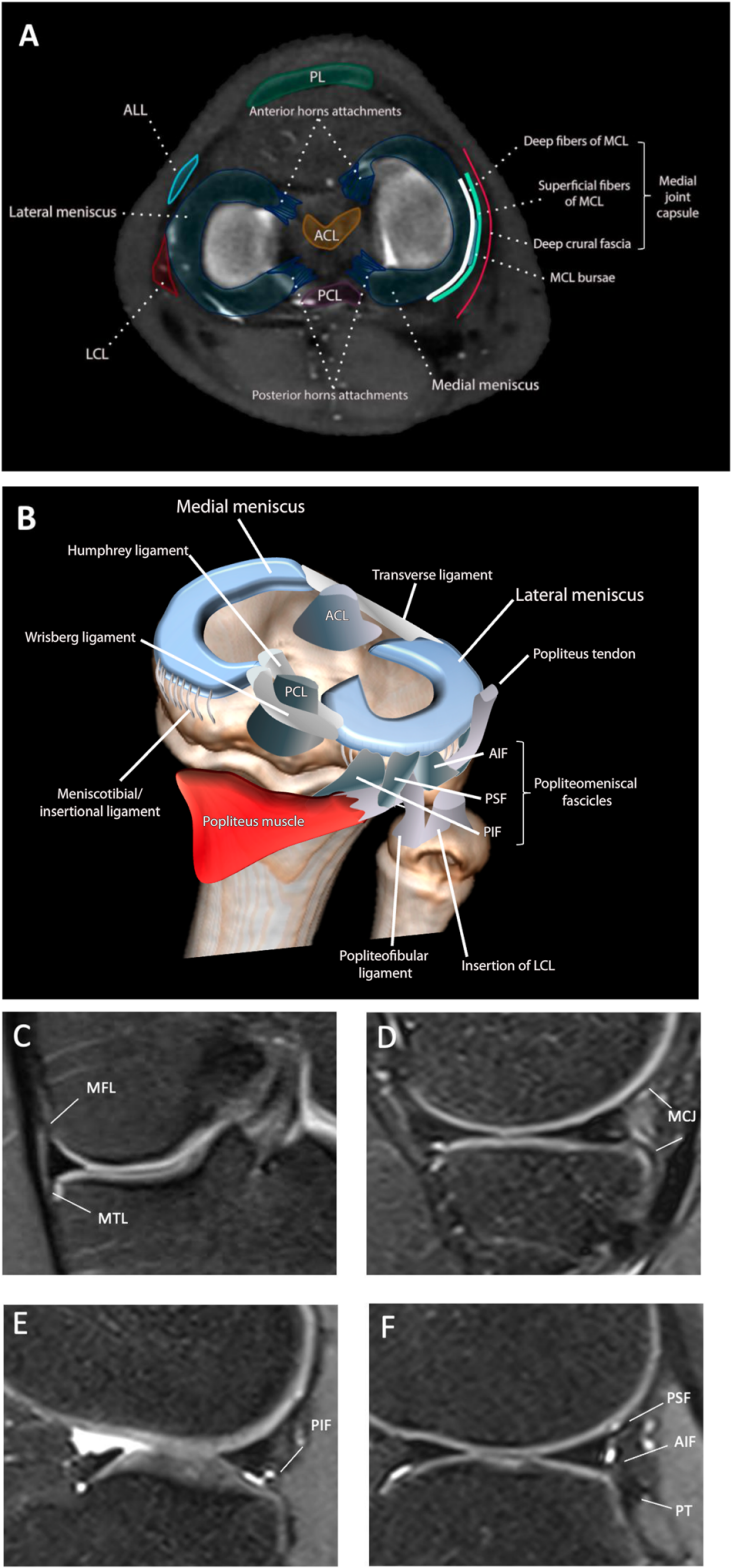
Illustration showing the anatomy of the knee in the axial plane of the MR image (**A**), demonstrating the meniscal roots and the relationship of the menisci with the local capsuloligamentous structures. **B** Three-dimensional schematic drawing with a posterolateral view of the meniscal anatomy, with a focus on the ligamentous structures. The MR images (**C**–**F**) include coronal (**C**) and sagittal (**D**) T2 FS-weighted images of the MM, highlighting the meniscofemoral, meniscotibial, and meniscocapsular junctions. Sagittal views of the LM (**E**, **F**) display the popliteomeniscal fascicles and the popliteus tendon. ALL, anterolateral ligament; PL, patellar ligament; MCL, medial collateral ligament; ACL, anterior cruciate ligament; PCL, posterior cruciate ligament; LCL, lateral collateral ligament; AIF, anteroinferior fascicle; PSF, posterosuperior fascicle; PIF, posteroinferior fascicle

The menisci are two crescent-shaped fibrocartilaginous structures situated between the femoral condyles and tibial plateaus that act as shock absorbers, dissipating stress and dispersing compressive loads [6]. The medial meniscus (MM) typically has a C-shaped morphology and is in close contact with the surrounding capsule. The lateral meniscus (LM) is almost uniformly circular, and in contrast to the MM, it is smaller and has increased mobility due to fewer capsular attachments [6]. The meniscotibial ligaments, also known as the coronary ligament, are a band of fibrous tissue that circumferentially anchors the peripheral margin of the medial and lateral menisci to the edge of the tibial condyle and functions to provide stability to this complex [7]. The meniscotibial ligaments are relatively thicker and stronger on the medial side of the knee and are particularly thick over the segment of the meniscal body, where they lie deep to the superficial medial collateral ligament, and in the posterior segment of the posterior oblique ligament [8]. The lateral meniscotibial ligament is thinner and more elastic relative to the medial side, lacks the thickenings observed on the medial meniscotibial ligament, and is also absent throughout the popliteal hiatus. These features make the LM intrinsically more mobile than the MM [8]. Additional attachments to the LM include the meniscofemoral ligament, popliteomeniscal fascicles, and meniscofibular ligament. Two meniscofemoral ligaments exist: the anterior meniscofemoral ligament (aMFL), referred to as the ligament of Humphrey, and the posterior meniscofemoral ligament (pMFL), known as the ligament of Wrisberg. These ligaments link the posterior horn of the LM to the lateral aspect of the medial femoral condyle. The aMFL travels anteriorly to the posterior cruciate ligament (PCL), whereas the pMFL courses posteriorly to the PCL [9] (Fig. 1). Biomechanically, these ligaments act as stabilizers of the posterior horn of the LM at various points throughout the range of motion. They additionally contribute to reducing anterior–posterior laxity of the knee by directing the posterior horn of the LM anteriorly and medially during flexion. Furthermore, the meniscofemoral ligaments play a significant role in resisting the posterior tibial drawer in both intact and PCL-deficient knees, particularly between 15° and 90° of flexion [9].

The popliteomeniscal fascicles arise from capsular extensions that blend with the popliteus musculotendinous junction and attach to the peripheral posterior horn of the LM. These fascicles consist of the anteroinferior, posterosuperior, and posteroinferior components. Damage to the attachments of the popliteomeniscal ligament complex has been reported to result in meniscal hypermobility and subluxation, often occurring concomitantly with ACL and posterolateral corner injuries [10]. The meniscofibular ligament originates from the LM and inserts into the fibular head, just anterior to the popliteus muscle origin [8]. There is no significant anatomical difference in the overall configuration or attachments between the pediatric and adult meniscus, although developmental changes in tissue composition may occur with age.

## Meniscal development, vascularity, and histology

The development of menisci occurs through the differentiation of mesenchymal tissue located in the lower limb bud. They appear during the fourth week of human development, become evident at 9 weeks, and assume an adult-like shape form at 14 weeks of gestation [11]. Blood supply arises peripherally from a perimeniscal capillary plexus. The entire meniscus is vascularized during the fetal period and at birth. By 9 months of age, vascular regression of the inner one-third has occurred [12]. The intrameniscal vascularity gradually decreases centrally, and at more than 10–12 years of age, the menisci have only peripheral vessels (10–30% of the meniscus), like the adult pattern. The peripheral red zone has relatively good healing potential because of its rich blood supply. The white zone corresponds to the inner 70–90% and is essentially avascular, which is nourished only by passive diffusion and heals poorly; thus, tears in this area tend to propagate and usually require intervention. In between these zones, there is a middle transitional zone (termed the red–white zone) with intermediate vascularity [13]. High signal intensity in the menisci of children may reflect normal vascularity, which decreases with age. When it is linear, it may be confused with a meniscal tear, but the vascularity should not contact the articular surface [14]. Before 3 months of age, the menisci predominantly contain cells with high cytoplasm‒to‒nucleus ratios, which decrease between 3 months and 9 months of age, whereas the amount of collagen increases. From 3 years of age onward, the ultrastructure of the menisci begins to transform, with the majority of collagen fibers arranged circumferentially, parallel to the long axis of the meniscus. As a child grows, becomes more erect, and starts to walk, more circumferential fibers and, occasionally, radial fibers can be observed near the articular surface. This process is completed around the age of 9 years, when the menisci are predominantly composed of collagen rather than fibroblasts. The ultrastructure of the meniscus in children aged 10 years or over is similar to that in adult meniscal tissue and consists mainly of type I collagen fibers arranged circumferentially parallel to the long axis, with relatively few fibroblasts observed [13].

## Anatomical variants

### Discoid meniscus (DM)

A DM is a congenital abnormality of the morphology of the meniscus, in which the meniscus has the appearance of a flat disk instead of the usual semilunar shape. Discoid morphology occurs most commonly in the LM, although cases involving the medial compartment have also been described [15].

The incidence of this condition is estimated to be 0.4–17% for the lateral DM and 0.06% for the medial DM, and it is bilateral in 20% of cases [16]. However, the true incidence and prevalence are limited, as many discoid menisci are asymptomatic and incidentally detected [17].

The clinical presentations of discoid menisci can vary significantly, ranging from no symptoms to severe pain during movement. In adults, the commonly observed symptoms include pain during motion, joint locking, instability (giving way), and the classical “popping knee” syndrome (a snapping sound heard from the affected knee) [18]. These symptoms are primarily associated with meniscal tears, detachment, or defects in the pMFL.

However, in children, the main clinical manifestations of DM differ from those in adults. Notable symptoms in children include extension block, knee pain, joint line tenderness, locking, clicking, and giving way [19].

Extension block, which is rarely observed in adults, is the primary clinical manifestation of DM without a tear in children. This may be due to the position or morphology of discoid menisci in developing knee joints or to the relatively large volume of the discoid lateral meniscus (DLM) occupying the smaller inner space of the knee joint. There appears to be a close relationship between extension block and meniscal morphology, especially the thickness of the anterior part of the meniscus, which is significantly greater than the posterior thickness in children with extension block [19].

Magnetic resonance imaging (MRI) has proven useful for diagnosing a DLM. In adults, the diagnosis of DM is traditionally made if three or more 5-mm-thick contiguous sagittal images show continuity of the meniscus between the anterior and posterior horns [19]. Additionally, Araki et al [20] reported that a DLM is present if the transverse width at the mid-portion of the meniscal body exceeds 14 mm, regardless of the tibial width [20].

**Fig. 2:**
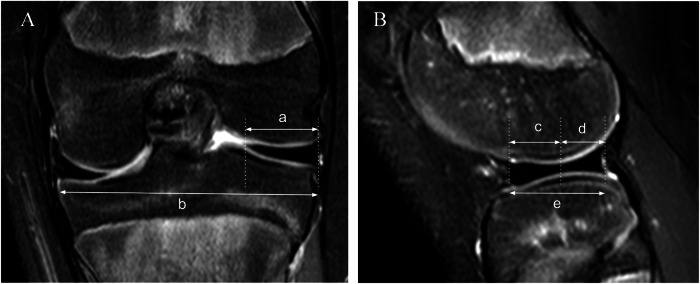
Images of MRI measurements obtained from a child presenting with an incomplete DLM. **A** Coronal slice. Meniscal width (a) and maximum tibial width (b). The RMT to the tibia: a/b x 100%. **B** Sagittal slice. Width of the anterior and posterior horns (c and d, respectively) to the maximum meniscal diameter (e). Percent coverage of the meniscus: (c + d)/e × 100%

In a study that included children in the population, a new diagnostic criterion for DM was described. The most accurate diagnostic criteria were either a ratio of the meniscus (RMT) to the tibia ≥ 20% or a percent coverage of the meniscus (PCM) ≥ 75%. The RMT is the ratio of the minimum meniscal width to the maximum tibial width on the coronal slice, and the PCM is the ratio of the sum of the width of the anterior and posterior horns to the meniscal diameter on the sagittal slice showing the maximum meniscal diameter. Both ratios have a sensitivity and specificity of 95% and 97%, respectively, even in the presence of torn menisci (Fig. 2) [21].

**Fig. 3:**
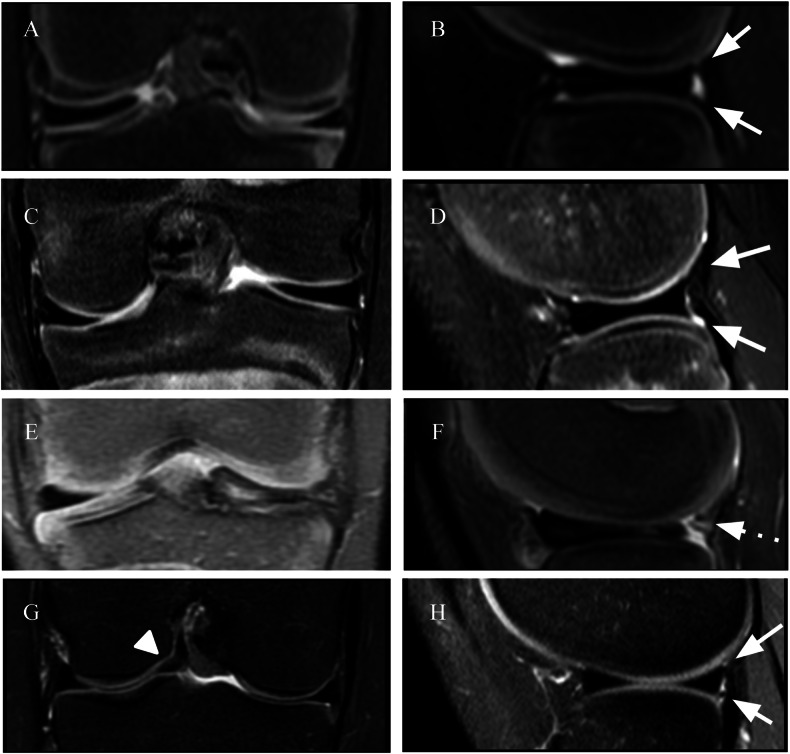
Watanabe classification. Type I (**A**, **B**): complete discoid with a normal posterior attachment (arrow). Type II (**C**, **D**): incomplete discoid, with a normal posterior attachment (arrow). Type III (**E**, **F**): Wrisberg variant with instability due to a lack of the usual posterior attachment (dashed arrow). Proposed type IV (**G**, **H**): ring-shaped morphology of the meniscus with triangular meniscal tissue on the central portion of the lateral compartment (arrowhead) and a normal posterior tibial attachment (arrow)

**Fig. 4:**
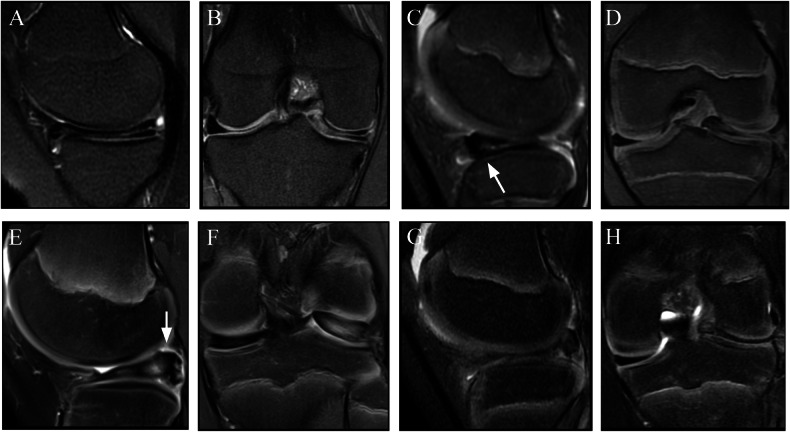
**A**, **B** Medial DM with an extensive horizontal tear extending to the inferior articular surface and a small perimeniscal cyst. No shift. **C**, **D** Anterocentral shift. The meniscus is dislocated forward (arrow), with a phantom posterior segment aspect. **E**, **F** DM with posterior displacement of the LM (arrow) and posterocentral shift. **G**, **H** Central shift. The meniscus is dislocated inward

A DM is at a biomechanical disadvantage because of factors such as shape, ultrastructure, and stability. The DLM is thicker and has poor vascularity in the inner areas [22]. The collagen fibers in the DM are disorganized and decreased in number. A lower collagen concentration, similar to degenerated menisci, may increase the vulnerability of the meniscus. Loss of organization may decrease the capacity of the menisci to dissipate hoop stresses, which may predispose the menisci to tears [23]. The most common tear pattern of the DM is a degenerative horizontal cleavage tear (43%) [24]. Multiple classification systems of the DM have been proposed, with the most commonly used being that proposed by Watanabe et al in 1978 [24, 25]. They described three major meniscal abnormalities: (type I) complete, disk-shaped meniscus with a thin center covering the tibial plateau; (type II) incomplete, semilunar-shaped meniscus with partial tibial plateau coverage; and (type III) Wrisberg type, a hypermobile meniscus that lacks attachments to the capsule through the normal fascicles and to the tibia through meniscotibial ligaments. It is important to carefully evaluate whether the DM corresponds to the Wrisberg type, as it has particular prognostic implications. This subtype has a greater chance of subluxation during knee flexion because of its attachment to the posterior capsule solely through the Wrisberg ligament. Additionally, on MR images, the lack of these normal fascicles may appear as a high T2 signal interposed between the LM and the joint capsule, simulating a peripheral tear or a fascicle lesion (Fig. 3). Recently, a fourth type, characterized by a ring-shaped meniscus, has been proposed [26, 27]. A ring-shaped meniscus is a variant characterized by a ring-shaped morphology with a normal posterior tibial attachment. Ahn provided an MRI classification using a standard knee protocol for DM that does not aim to describe the type of lesion but instead shows the resulting meniscal displacement. There are 4 described types: (1) No shift: the peripheral portion of the DM is not separated from the capsule, and the entire meniscus is not displaced; (2) anterocentral shift: the periphery of the posterior horn is detached from the capsule, and the entire meniscus is displaced anteriorly or anterocentrally; (3) posterocentral shift type: the periphery of the anterior horn is detached from the capsule, and the entire DM is displaced posteriorly or posterocentrally; (4) central shift type: the periphery of the posterolateral portion is torn or lost, and the entire DM is displaced centrally toward the intercondylar notch (Fig. 4) [28].

Symptomatic discoid menisci with evidence of tears or instability should be treated. Therefore, an accurate diagnosis and appropriate treatment are essential for patients with symptomatic DM [28].

#### DLM instability

**Fig. 5:**
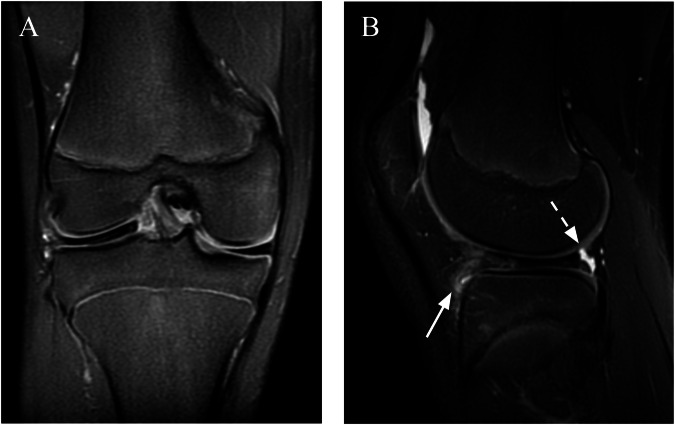
A 13-year-old boy complained of pain during running and leg extension movement. **A** Coronal and (**B**) sagittal FS T2-W MR images of the right knee show no shift-type complete lateral degenerated DM, parameniscal soft-tissue edema (arrow), and absence of the posterosuperior popliteomeniscal fascicle (dotted arrow). These are signs of PRI

**Fig. 6:**
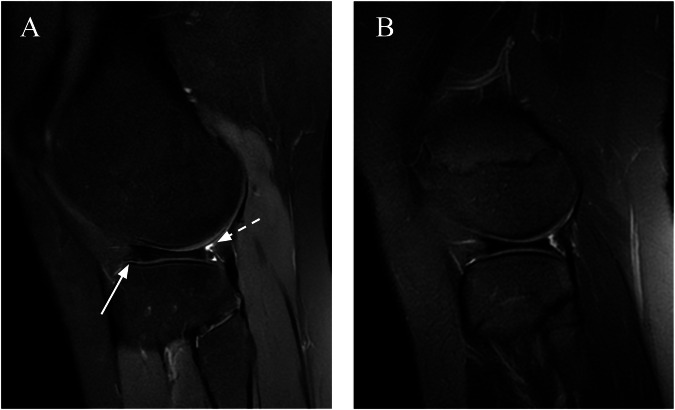
A 20-year-old woman underwent a follow-up examination of the DM. **A** Sagittal FS T2-W MR image of the right knee showing anterior bulging of the meniscal margin that was not evident in a previous exam (**B**) performed 5 years before. Note, the absence of the posterosuperior popliteomeniscal fascicles (dotted arrow) (Wrisberg type). These signs together suggest PRI

In an arthroscopic study, the rate of instability among symptomatic discoid lateral menisci was 77%, with anterior instability present in 53% of cases and posterior instability present in 16% of cases. Instability is defined as evidence of hypermobility and peripheral detachment of the remnant meniscus assessed by systematic probing of the meniscus after saucerization [24]. Preoperative MRI can reveal peripheral rim instability (PRI) by showing the shift mentioned above (anterocentral, posterocentral, central shift). Some no-shift-type complete discoid lateral meniscal lesions may be classified as stable on the basis of MRI findings but may have PRI during arthroscopy. Some predictive MRI signs of PRI in patients with the no-shift type of complete DLM have been described which may be useful in determining the location of the PRI. Anterior parameniscal soft-tissue edema and linear fluid signal at the anterior meniscal margin have high positive predictive value (PPV), sensitivity, and specificity in predicting anterior PRI. Bulging of the meniscal margin and the absence of the popliteomeniscal fascicle had high sensitivity but low PPV and specificity in predicting posterior PRI [29]. Therefore, preoperative MRI prediction of the presence and location of the PRI could help surgeons identify meniscal instability before surgery (Figs. 5 and 6).

#### Hypermobile LM

Hypermobility of the LM has been described in the pediatric literature [30, 31] and is characterized by lateral knee joint line pain with locking during deep flexion in the absence of a meniscal tear or discoid morphology [32]. In most cases, there is an abnormal anterior translation of the posterior horn of the LM during flexion, which is reduced in extension [33]. One case of a hypermobile anterior horn of the LM has been reported [34]. In children, the cause tends to be congenital deficiency of the posterior capsular attachments, whereas in adults, it is more likely to be traumatic [32]. The hypermobile LM can be confirmed via arthroscopy by translation beyond the midpoint of the lateral condyle or tibia using a probe [33].

Most patients with hypermobile lateral menisci reported in the literature received negative MR results of their lateral menisci on initial review, and in some cases, patients able to reproduce locking were scanned in the locked position, allowing verification of meniscal displacement [35].

A study by Suganuma in patients with mechanical locking and arthroscopically proven lateral meniscal subluxation revealed that inspection of MR images would show abnormalities in the popliteomeniscal fascicles in most cases, such as an unclear band running from the superior (or inferior) border of the LM or the absence of a visible band [36].

LaPrade et al described a provocative maneuver known as the Figure 4 test which places the affected knee in flexion, varus, and external rotation. This test may reproduce locking in patients with a hypermobile LM [37].

Therefore, in the context of clinical suspicion of hypermobile lateral menisci, the radiologist should be aware and perform a close inspection of the lateral meniscal capsular attachments, and potentially perform additional sequences with a provocative maneuver.

#### Other meniscal variants

**Fig. 7:**
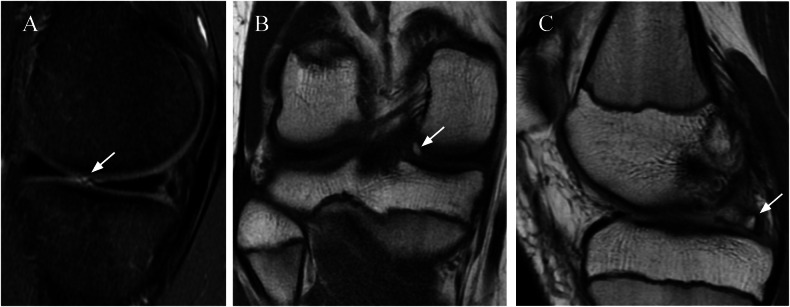
Other meniscal variants. **A** Sagittal FS T2-W image showing the typical rippled (arrow) appearance of a meniscal flounce. Potential pitfalls may simulate a truncated meniscus and mimic a radial tear. **B**, **C** A 14-year-old male with a previous tear of the posterior root of the MM (not shown), presented with a posttraumatic meniscal ossicle (**B**) Coronal T1 (**C**) and sagittal DP images show a meniscal ossicle within the posterior root of the MM (arrows). The ossicle can mimic a tear, which is a potential pitfall

Meniscal flounce is typically secondary to flexion of the knee and redundancy of the free edge of the MM, which is observed in 0.2–0.3% of asymptomatic knees. Classically described as an anatomical variation, some recent studies have shown an association with medial meniscal tears (Fig. 7A). The meniscal ossicle is a small focus of ossification within the meniscus, usually the posterior horn of the MM. This may represent an embryologic variant or sequelae of prior trauma. Meniscal ossicles are rare and are often clinically silent but may cause mechanical symptoms or pain and can easily be mistaken for a loose body on radiographs or MRI (Fig. 7B, C) [38]. Congenital hypoplasia and absence of the meniscus are extremely rare.

## Diagnosis

The clinical diagnosis of meniscal injury in children is difficult, as the following signs are nonspecific and nondiscriminating: joint pain, and internal derangement of the knee with cracking and/or locking. Clinical examination is currently systematically associated with MRI [39]. MRI is widely used for the evaluation of the knee in children because it has many advantages, such as a lack of ionizing radiation; multiplanar imaging capabilities; excellent resolution; and superior evaluation of soft tissues, bone marrow, cartilage, muscle, ligaments, and tendons [40]. Furthermore, in most cases, sedation is not needed in children [12].

MRI is the imaging modality of choice for diagnosing meniscal injury; however, diagnosing pediatric meniscal tears can be challenging, as discoid meniscal tears and lateral meniscal tears may be relatively often missed (26.7% and 18.8%, respectively) [41]. Furthermore, compared with diagnostic arthroscopy, MRI overestimates medial meniscal lesions and underestimates lateral meniscal lesions [39]. This may be explained by the increased vascularity of the meniscus in children, which manifests as a horizontal linear high signal on T2-weighted images and may obscure or mimic a tear [42]. Likewise, a linear hypersignal in a DM does not systematically indicate a lesion [43]. In adults, the global sensitivity and specificity of MRI for meniscal tears are 92.0% and 90.0% for medial meniscal tears and 80.0% and 95.0% for lateral meniscal tears, respectively [44]. Few pediatric studies have assessed the precision of MRI for this purpose [2, 39, 45–47]. In 2001, Kocher et al reported that MRI had decreased sensitivity (62%) and specificity (90%) in children (< 12-years-old) than in adolescents (12–16-years-old) [47]. High-quality images are important for evaluating menisci, especially in children, optimizing the protocol and using the proper coil are essential for obtaining a high-quality image in the shortest possible time.

## MRI protocol

Conventional MRI without intraarticular contrast provides a noninvasive assessment of both pre- and postoperative pediatric knees in most cases, though it has lower sensitivity, specificity, and accuracy compared to direct or indirect MR arthrography for detecting meniscal retears. MRI is often used to evaluate unresolved or recurrent knee pain following meniscus surgery or to assess the status of the operated meniscus. Regardless of the imaging protocol, key information such as the patient’s baseline history, symptoms, operative details (type and date of surgery), and preoperative imaging for comparison should be included, as direct and indirect MR arthrography are shown to have similar and superior accuracy to conventional MRI in detecting meniscal retears [48].

In our practice, we routinely begin both pre- and postoperative imaging with conventional MR imaging. Our preference is to start with a screening examination of paired fast spin‒echo (FSE) proton density (PD) and fat-suppressed T2 images. In postoperative cases, if there are substantial postoperative artifacts, we perform a metal-artifact reduction protocol that includes optimized FSE PD and FSE inversion recovery pulse sequences. We also include an isotropic 3D FSE sequence in all pediatric knee MRI studies. Reconstructions (especially radial) of 3D FSE sequences perpendicular to the course of the menisci and at the site of surgery can supplement the assessment and characterization of meniscal lesions, especially in regions that are suboptimally assessed in standard planes [49]. The complete MRI sequence parameters are shown in Table 1. In our institution, arthrography techniques are reserved for uncommon cases in which additional problem-solving is deemed necessary. A previous study in adults showed that postsurgical assessment is also most often performed without contrast [50]. It is important to recognize that arthrography techniques may be associated with additional time, cost, discomfort, family and patient stress, and risks such as urticaria, vasovagal and anaphylactic reactions, and septic arthritis, especially in pediatric patients. Additionally, in several countries, the intra-articular administration of gadolinium-based contrast agents is off-label.

**Table 1:** MRI sequence parameters

	TR, (ms)	TE, (ms)	BW, (kHz)	ETL	NEX	FOV, (cm)	Matrix	Slice/gap, (mm)	Time, (min)
3D FSE Fat Sat sagittal	1100	35	50	32	2	16	384 × 384	1/NA	05:55
FSE DP Sagittal	2500	20–30	62.5	13	1	16	512 × 480	2.2/0.4	01:48
FSE T2 Fat Sat axial	2500	40–50	62.5	13	1	16	512 × 480	3.5/0.3	01:50
FSE T2 Fat Sat coronal	2500	40–50	62.5	13	1	16	512 × 480	3.5/0.3	01:08
T1 coronal	750	Minimum	41	13	0.5	16	512 × 416	3.5/0.3	00:55

*TR* repetition time, *TE* echo time, *BW* bandwidth, *ETL* echo train length, *NEX* number of excitations, *FOV* field of view, *Fat Sat* fat saturation

## Meniscal injuries

**Fig. 8:**
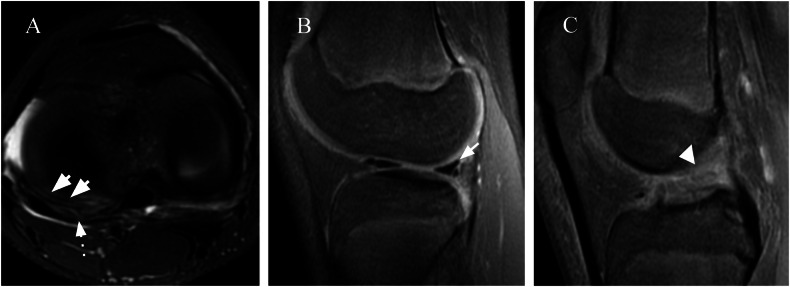
A 12-year-old female with a history of knee sprain evolving with pain and movement limitation. **A** Axial T2FS image showing a meniscal tear on the posterior horn (dotted arrow) and signs of perimeniscites (arrow). **B**, **C** Sagittal T2FS image showing an ACL tear (arrowhead) and a vertical tear on the posterior horn of the LM (arrow)

**Fig. 9:**
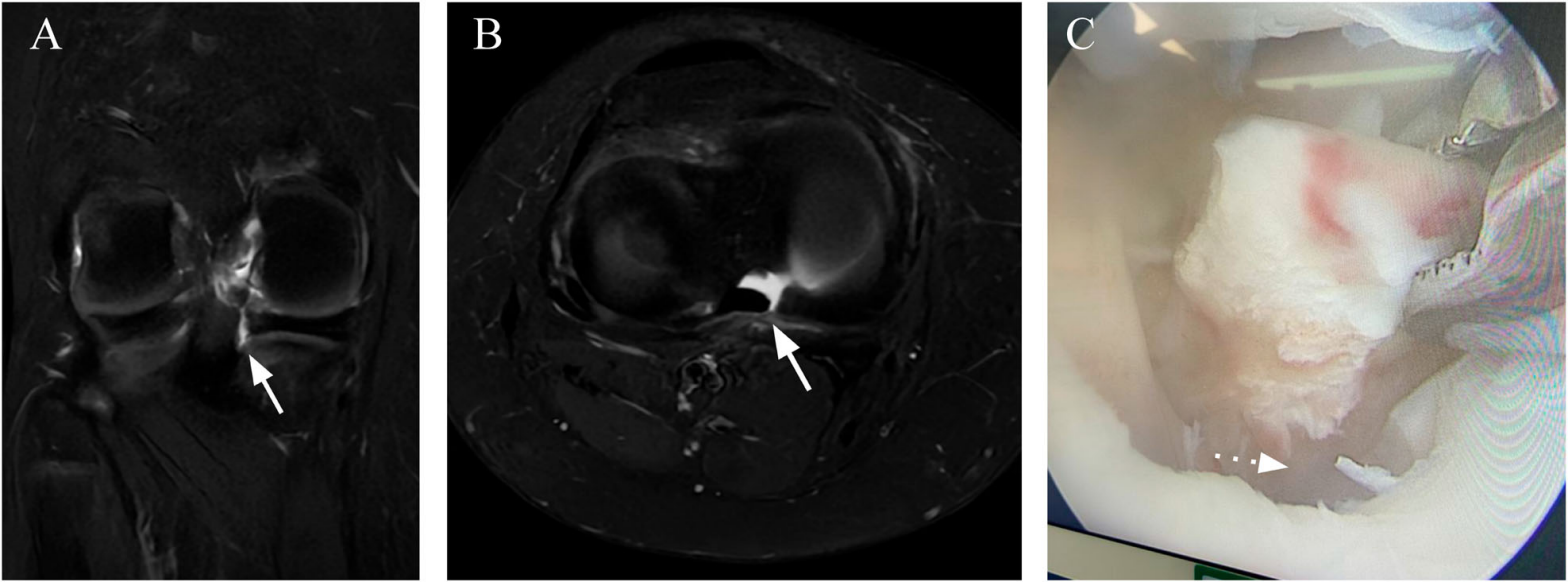
A 9-year-old female with a history of knee pain after falling on roller skates. **A** Coronal and (**B**) axial FS T2-W MR images of the right knee showing an MM posterior to the radial root tear (arrow). **C** Arthroscopy performed a few days later showed a complete radial root tear (dotted arrow)

Meniscal injury is one of the most commonly reported internal derangements in skeletally immature knees; however, the incidence of meniscal tears is significantly lower than that in the adult population [2]. In children, it is important to distinguish whether meniscal injury occurs in a normal or abnormal structure, such as DM. Tears in the DM are not necessarily associated with acute trauma and have a lower mean age (12.7) than nondiscoid tears (15.7) [3, 43]. Injuries in the normal meniscus are usually secondary to sports trauma and are rare in individuals under 10 years of age. The lesions may be isolated or, more often, associated with knee instability and are the same type as those seen in adults but in different proportions [43]. Some studies have shown that meniscus tears more often affect the LM than the MM in young patients under 20 years of age. Additionally, the prevalence of isolated medial meniscal tears increases with age [3, 51]. Isolated meniscal tears are more common in the younger pediatric population, whereas older adolescents are more likely to have a concomitant ligamentous injury, which might be explained in part by increased participation in competitive sports in that age group. The most common associated injury among high school athletes is ACL lesions (36.9%) [4]. In a study by Samora et al [52] with a total of 124 skeletally immature patients who underwent arthroscopic ACL reconstruction, the meniscal injury was associated with acute ACL rupture, with a prevalence of 69.3%. The LM was involved exclusively in 41% of the patients, whereas the MM was involved exclusively in 13% of the patients; both menisci were involved in 15% of the patients. The main meniscal tear patterns in this study were vertical and peripheral [36], similar to our case presented in Fig. 8. According to Shieh et al a retrospective study of 293 children and adolescents with meniscal tears who underwent arthroscopic surgery, the most common tear pattern was complex (28%), followed by vertical (16%), discoid (14%), bucket-handle (14%), radial (10%), horizontal (8%), oblique (5%), fray (3%), and root detachment (2%). Additionally, complex tears were associated with male sex and a higher body mass index. The posterior horns of the menisci are most commonly involved [3]. Although meniscus root tears are not common in the pediatric population, an undiagnosed lesion may lead to catastrophic consequences, as it profoundly affects meniscal biomechanics and is a precursor to joint damage and accelerated osteoarthritis (OA) [53]. Unlike in adults, in which tears of the MM posterior root are more common, in younger patients, meniscus root tears most commonly occur as root avulsions of the posterior root of the LM and are associated with ACL injury [54]. In our patient (Fig. 9), a medial meniscal root tear occurred without ACL injury. Immediate diagnosis is particularly important in pediatric patients because age- and location-dependent meniscal vascularity makes acute tears of the posterior root more amenable to repair and potential healing [55].

### Meniscal tear: What to report?

An accurate description of meniscal tears is very important. A radiological report must include whether there is any anatomical meniscal variation and the type, location, and extent of the meniscal tear, including which surface the tear extends to (femoral surface or tibial surface). Additionally, it is important to report any signs of instability and seek associated injuries. A clear description may obviate unnecessary surgery or may enable better surgical planning [56].

The traditional description of a meniscal tear is that of a linear increased signal intensity within the meniscus that extends to the femoral or tibial surface. In terms of orientation, a meniscal tear can be horizontal, radial, longitudinal, bucket handle, or complex (Table 2). A horizontal tear extends through the meniscus along a plane parallel to the tibial plateau, separating the meniscus into superior and inferior fragments. Radial tears are perpendicular to the long axis of the meniscus and begin in the free edge of the meniscus. A longitudinal tear extends through the meniscus parallel to its long axis in a vertical orientation and away from the free edge of the meniscus. If the tear extends over a long enough distance, the inner fragment may become displaced into the intercondylar notch, and it is then referred to as a bucket-handle tear. A complex tear involves a signal abnormality contacting the meniscal surface in more than one orientation, creating more than one tear fragment [56–58]. In addition to abnormal signal intensity, a tear may manifest as abnormal meniscal morphology, such as irregularity of the meniscal margin, a focal defect in one of its articular surfaces, or an abnormally small meniscal segment. If a segment smaller than the normal meniscal segment is observed, a thorough search should be performed to identify a displaced fragment [58].

**Table 2:** Meniscal tears: what to report

What to report	
SHAPE OF THE MENISCUS	Normal
	Discoid
TYPE OF THE TEAR	Horizontal
	Radial
	Vertical
	Longitudinal
	Bucket-handle
	Complex
LOCALIZATION	Peripheral
	Near to free edge
TEAR LENGTH	< 2.5 cm (good prognosis)
	> 2.5 cm
SINGS OF HYPERMOBILITY/ INSTABILITY	Meniscal displacement
	Lack of the usual posterior attachment in LM
	Meniscocapsular junction injury
ASSOCIATED INJURIES	Ligaments
	Cartilage and subchondral bone

*LM* lateral meniscus

The localization of the tear is important to describe, as it may change the therapeutic plan. A peripheral tear is confined to the outer third of the meniscus and is more likely to heal with conservative therapy or operative repair because of the rich vascularity [17]. Ramp lesions occur when the meniscus separates from the adjacent capsule and can lead to meniscal instability. This injury is most commonly observed along the MM, which is more tightly adherent to the joint capsule but can also occur along the posterolateral corner of the joint where the LM is attached to the popliteal meniscal fascicles. In pediatric patients, MRI findings suggestive of ramp lesions include medial meniscal tears, peripheral meniscal irregularities, fluid-like signal intensity at the meniscocapsular junction, and capsular ligament tears [59].

## General treatment concepts

The treatment of meniscal tear injuries in children and adolescents focuses primarily on meniscal repair. Other less usual treatment options include conservative treatment, meniscectomy, or replacement (meniscal scaffold or meniscal allograft transplantation).

Conservative treatment may be indicated for asymptomatic meniscal tears and an asymptomatic DM [60]. Some small, nondisplaced meniscal tears in the outer vascular region may heal spontaneously or become asymptomatic. Nonoperative treatment consists of rehabilitation of the damaged knee while avoiding pivoting and sports for 12 weeks [61].

Most meniscal tears in pediatric patients are large and require surgical treatment. Meniscal repair can potentially diminish the risk of future degenerative joint changes and is strongly indicated in cases of middle one-third and outer one-third tears (vascular zone), longitudinal vertical tears, and concomitant meniscus repair with ACL reconstruction [60, 62]. A systematic review revealed that meniscal repair performed in this population was associated with good to excellent outcomes, low complication rates, and high clinical evidence of healing, considering isolated meniscal repairs or in combination with concomitant ACL reconstruction [63]. Conversely, the reported outcome of total or subtotal meniscectomy in the pediatric population is poor and could lead to greater OA than in adults [60].

The need for meniscus replacement (meniscal allograft transplantation) in children is rare and a viable option in the pediatric population with post-meniscectomy syndrome [60].

## Postoperative MRI evaluation

After meniscal surgery, meniscal morphology may differ from that of a nonoperative meniscus. Fluid-signal intensity extending through the site of repair on T2-weighted images may be considered a normal finding following meniscal repair. Surfacing intrameniscal signal changes in this setting may be due to immature fibrovascular granulation tissue and mature fibrocartilaginous scar tissue at the repair site that may persist on MR imaging for 6 months to up to 12 years following successful meniscal repair [64].

Specific findings of the retorn meniscus following meniscal repair or partial meniscectomy are displaced meniscal fragments, increased signal intensity extending through the site of repair on T2-weighted images, and abnormal signal intensity at a site distant from the repair site [64].

Direct MRI arthrography can be used when conventional MRI does not reveal the underlying cause of the patient’s symptoms or when there is strong clinical suspicion of a recurrent or residual tear, especially in the absence of preoperative MRI for comparison [65]. A significant imaging finding in MRI arthrography is contrast material tracking into the tear on T1-weighted sequences, which serves as a reliable indicator of a meniscal retear [66].

**Fig. 10:**
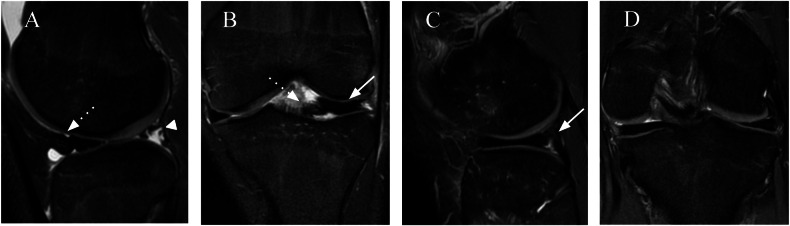
A 17-year-old male soccer player with a history of knee trauma and postsurgical suture evaluation 6 months later. **A**, **B** Sagittal and coronal T2FS: presurgical MR image showing detachment of the posterior horn of the meniscocapsular junction (arrowhead), anterior displacement of a portion of the meniscus (dotted arrow), and thickening of the anterior horn (arrow). **C**, **D** Sagittal and coronal T2FS: image 6 months after meniscal surgical suture showing posterior meniscocapsular repair (arrow) with good evolution and an appearance similar to that of the contralateral meniscus shown in **D**

**Fig. 11:**
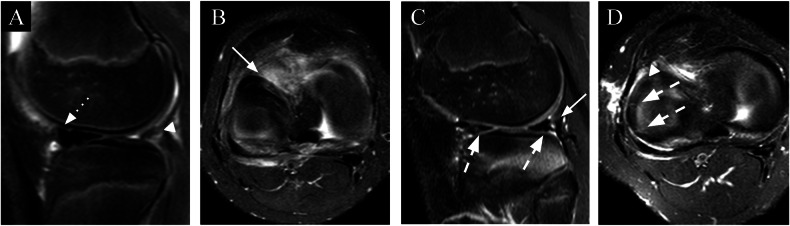
An 11-year-old male with a history of knee trauma and postsurgical suture evaluation 2 months later. **A**, **B** Sagittal and axial T2FS: presurgical MR image showing detachment of the posterior horn of the meniscocapsular junction (arrowhead) with anterior displacement (dotted arrow) and thickening of the anterior horn (arrow). **C**, **D** Sagittal and axial T2FS: image 2 months postsurgical suturing of the meniscus with reinsertion of the posterior horn showing posterior meniscocapsular repair (arrow) and high signal lines within the body and anterior and posterior horns indicating the suture sites (dashed arrow)

**Fig. 12:**
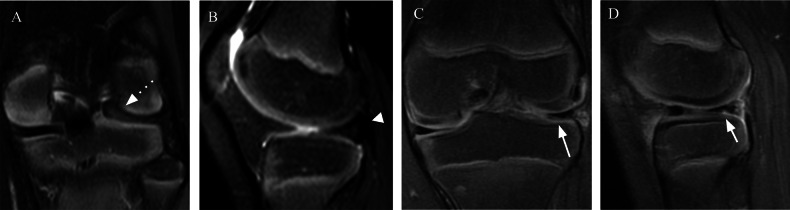
A 7-year-old female with a history of knee pain after trauma and a postsurgical evaluation 3 months later. **A**, **B** Coronal and sagittal T2FS: presurgical MR image showing a posteriorly subluxated DM (arrowhead) due to a horizontal tear (dotted arrow) with separation of the anterior meniscocapsular junction (not shown) and a posterocentral shift. **C**, **D** Coronal and sagittal T2FS: meniscus image 3 months after surgery showing a volumetric reduction, contour irregularities, and shortening/amputation of the free margins and foci of the intrasubstantial signal changes related to previous manipulation (arrows)

Figures 10–12 show images of patients undergoing evaluation for postsurgical assessment of the meniscus.

## Conclusion

Meniscal tears in children and adolescents are not uncommon; thus, it is important for radiologists to be familiar with normal and abnormal appearances of the menisci on MRI. Knowledge of the particularities of the pediatric meniscus, including the epidemiology of meniscal tears, anatomical variations, meniscal vascularization, and associated lesions, is essential for precisely describing meniscal tears.

Given its greater blood supply, the meniscus in skeletally immature children may have better reparative potential than the adult meniscus. Therefore, meniscal repair of this injury usually results in good to excellent outcomes.

## Abbreviations

ACL: Anterior cruciate ligament
aMFL: Anterior meniscofemoral ligament
DLM: Discoid lateral meniscus
DM: Discoid meniscus
Fat Sat: Fat saturation
FSE: Fast spin‒echo
LM: Lateral meniscus
MM: Medial meniscus
OA: Osteoarthritis
PCL: Posterior cruciate ligament
PD: Proton density
pMFL: Posterior meniscofemoral ligament
PPV: Positive predictive value
PRI: Peripheral rim instability
RMT: Ratio of the meniscus
